# Unmasking the hidden aftermath: postintensive care unit sequelae, discharge preparedness, and long-term follow-up

**DOI:** 10.62675/2965-2774.20240265-en

**Published:** 2024-05-27

**Authors:** Cassiano Teixeira, Regis Goulart Rosa

**Affiliations:** 1 Department of Internal Medicine Universidade Federal de Ciências da Saúde de Porto Alegre Porto Alegre RS Brazil Department of Internal Medicine, Universidade Federal de Ciências da Saúde de Porto Alegre - Porto Alegre (RS), Brazil.; 2 Department of Internal Medicine Hospital Moinhos de Vento Porto Alegre RS Brazil Department of Internal Medicine, Hospital Moinhos de Vento - Porto Alegre (RS), Brazil.

**Keywords:** Critical illness, Patient discharge, Hospital-to-home transition, Mental health, Cognition, Cardiovascular diseases, Intensive care units

## Abstract

A significant portion of individuals who have experienced critical illness encounter new or exacerbated impairments in their physical, cognitive, or mental health, commonly referred to as postintensive care syndrome. Moreover, those who survive critical illness often face an increased risk of adverse consequences, including infections, major cardiovascular events, readmissions, and elevated mortality rates, during the months following hospitalization. These findings emphasize the critical necessity for effective prevention and management of long-term health deterioration in the critical care environment. Although conclusive evidence from well-designed randomized clinical trials is somewhat limited, potential interventions include strategies such as limiting sedation, early mobilization, maintaining family presence during the intensive care unit stay, implementing multicomponent transition programs (from intensive care unit to ward and from hospital to home), and offering specialized posthospital discharge follow-up. This review seeks to provide a concise summary of recent medical literature concerning long-term outcomes following critical illness and highlight potential approaches for preventing and addressing health decline in critical care survivors.

## INTRODUCTION

The systematic organization of intensive care units (ICUs)^(1)^has played a pivotal role in guiding health care teams toward better outcomes in recent decades.^(2)^ Along with the rational use of resources^(3)^ and the protocolization of care,^(4,5)^a greater emphasis on humanization^(6,7)^ has been integral to this transformation, resulting in a reduction in short-term morbimortality among critically ill patients in recent years.^(8,9)^ However, this decline in mortality has brought to light a new challenge—the exacerbation of preexisting conditions or the development of new physical,^(10,11)^ mental,^(12)^ and psychological sequelae^(13)^among survivors. These disabilities might lead to reduced quality of life (QoL), increased health care expenditures, and a greater risk of rehospitalization and long-term mortality.^(14)^

Recently, professional society guidelines have addressed the importance of improving long-term outcomes among survivors of critical care.^(15)^ As a result, efforts are now underway to implement strategies within the ICU to prevent long-term disabilities,^(16)^ plan safe discharges to the ward and home,^(17,18)^ strengthen the continuity of care,^(19)^ and reduce ICU and hospital readmissions.^(20,21)^ In this context, discussions have progressed from theoretical considerations to tangible actions in the care of critically ill patients. The implementation of strategies for the comprehensive care of critically ill patients after their discharge from the ICU is now a priority,^(22)^and this shift toward a holistic approach can contribute to improving the overall well-being and long-term outcomes of critical care patients and their family members. This text refers exclusively to adult patients. Children were not considered in this review.

## OUTCOMES AFTER INTENSIVE CARE UNIT DISCHARGE

Patients who survive acute critical illness experience a significant level of morbidity^(23,24)^ and mortality^(25)^ in the months following their discharge from the ICU (Table 1). This risk is considerably greater than that in patients who do not require critical care,^(26)^ and it may persist at an elevated level for several years after hospital discharge, particularly in septic patients.^(27,28)^

**Table 1:** Sequelae of critical illness

Disorder	Consequences
Immunosuppression	Infections represent one of the most important reasons for readmission among critical patients, and a substantial portion of these could be averted. Furthermore, preventing readmission appears to reduce long-term patient mortality
ICU-acquired weakness	Prolonged mechanical ventilation, compromised ambulation, impaired ADL, pharyngeal muscle weakness, swallowing difficulties, increased risk of aspiration, employment difficulties, and/or reduced health-related quality of life for ≥ 5 years
Nutritional compromise	Compromised physical and neurocognitive recovery
Entrapment neuropathy	Foot or wrist drop, hindering rehabilitation and functioning
Frailty	Functional disability, new nursing home admission, increased post-ICU mortality
Cognitive dysfunction	Decreased attention, concentration, processing speed, memory, and/or executive dysfunction for ≥ 5 years; impact on employment and health status
Psychological disorders	Experiencing depressive symptoms, anxiety, PTSD, suicidality, and/or substance misuse for ≥ 8 years
Pressure injuries	Prolonged presence may impede return to work; associated with increased post-ICU mortality
Risk of falls	Falls can be potentially devastating for debilitated patients, and their prevention (e.g., muscle strengthening and educational measures) can significantly reduce morbidity
Oral complications	Gingivitis, dental caries, tooth injury or loss, requiring longer-term dental follow-up
Endocrinopathies	Disruption of thyroid, adrenal function, and hypothalamic–pituitary axis, affecting endocrine homeostasis and sexual function
Musculoskeletal disorders	Frozen joints, contractures, and heterotopic ossification
Changes in appearance	Alopecia, scarring, and disfigurement, affecting social reintegration
Taste changes	Difficulties with feeding and nutrition
Hearing or vision changes	Delayed recovery and return to home and work
Procedure-related trauma	Rectal and urethral injury, vocal cord dysfunction with altered phonation, tracheal stenosis, hindering ADL, rehabilitation, and return to home and work
Renal dysfunction	Chronic impairment of the glomerular filtration rate, necessitating renal-replacement therapy, compromising health-related quality of life, and leading to increased health care use
Cardiovascular disorders	Sepsis and COVID-19 survivors exhibit higher rates of cardiovascular and thromboembolic events

Source: Herridge MS, Azoulay E. Outcomes after critical illness. N Engl J Med. 2023;388(10):913-24.^(24)^

All postintensive care unit coexisting conditions have been qualitatively or quantitatively associated with long-term impairments in health-related quality of life, disruption of community integration, return to work, and increased health care costs.

ICU - intensive care unit; ADL - activities of daily living; PTSD - posttraumatic stress disorder.

### Higher long-term mortality

The mortality rates in the year following ICU discharge are high, but they vary significantly, ranging from 7% to 59%.^(28-35)^ Patients who have been through the ICU have 7% higher mortality rates after hospital discharge than those who were not admitted to the ICU.^(26)^ In the first months after ICU discharge, half of the deaths among patients are attributed to infectious complications, whereas the other half is linked to diverse factors, including advanced age, preexisting comorbidities, and poor functional status at the time of discharge.^(10,25)^

Numerous factors prior to ICU admission, such as age, comorbidities, sarcopenia, and frailty^(36-38)^ along with the severity of acute illness as measured by the number and degree of organ dysfunctions^(28)^ and acute ICU complications, such as muscle weakness, functional dependence, and psychological symptoms,^(39)^ seem to contribute nonlinearly to increased long-term mortality among ICU survivors. In particular, neurological dysfunctions, such as delirium and coma,^(40)^ and ICU-acquired muscle weakness,^(41)^ are the acute organ dysfunctions that have the most significant association with long-term mortality.

### Mental health impairment

The prevalence of prior psychiatric diagnoses is greater in critically ill patients than in hospital and general population cohorts.^(13)^ In the months after ICU discharge, approximately 32 - 40% of critical care survivors experience anxiety,^(42)^ 29 - 34% experience depression,^(43)^ and 16 - 23% experience posttraumatic stress disorder (PTSD).^(44)^ There is an increased occurrence of new psychiatric diagnoses and the use of psychoactive medications in the months following discharge.^(45)^ Within the first year postdischarge, nearly 20% of survivors start using new psychotropic medications (hypnotics, antidepressants, anxiolytics, or antipsychotics).^(46)^Moreover, there is a significant increase in the risk of self-harm and suicide among critical care survivors with prior psychiatric disorders.^(47)^Emotional distress and psychiatric morbidity following critical illness can be viewed as a collection of syndromes with overlapping symptoms and potential risk factors rather than completely distinct entities.^(13)^ Posttraumatic stress symptoms include hyperarousal, intrusive recollections, and avoidance behaviors associated with a traumatic event. Depression symptoms include a low mood, anhedonia (inability to experience pleasure), and feelings of guilt or worthlessness. Anxiety symptoms encompass excessive worry and feelings of dread or perceived threat. When these symptoms are significant and impact daily functioning, a diagnosis of a psychiatric condition can be considered.

**Figure 1:**
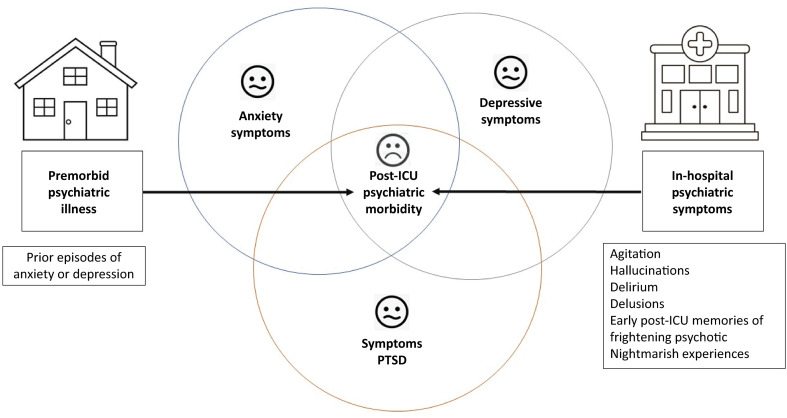
Potential risk factors for psychiatric morbidity following intensive care unit discharge include preexisting psychiatric illness and the presence of psychiatric symptoms during hospitalization.(13,48) The emojis represent the quality of life of patients based on the number of psychiatric morbidities. ICU - intensive care unit; PTSD - posttraumatic stress disorder.

Source: Adapted from: Prince E, Gerstenblith TA, Davydow D, Bienvenu OJ. Psychiatric morbidity after critical illness. Crit Care Clin. 2018;34(4):599-608^(13)^; Teixeira C, Rosa RG, Sganzerla D, Sanchez EC, Robinson CC, Dietrich C, et al. The burden of mental illness among survivors of critical care—risk factors and impact on quality of life: a multicenter prospective cohort study. Chest. 2021;160(1):157-64^(48)^.

The prevalence of mental health conditions tends to increase in the long term after ICU discharge,^(13)^ and many individuals experience a combination of psychiatric syndromes.^(13,48)^ The more psychiatric syndromes exhibited by a survivor, the greater the impact on their QoL,^(48-52)^and the greater their risk of mortality after leaving the ICU.^(53)^ However, identifying potential risk factors associated with mental illness among post-ICU patients is a complex undertaking that involves various factors across different domains, including age, preexisting mental health conditions, acute emotional stress, and physical impairment experienced during the ICU stay^(13,48)^ (Figure 1). Younger patients are at greater risk of developing PTSD, and a strong predictor of psychiatric illness related to postintensive care syndrome (PICS) or the PICS-family (PICS-F) is a previous history of mental illness.^(13,54)^

Family members of ICU patients also experience high frequencies of symptoms related to anxiety, depression, PTSD, and prolonged grief disorder.^(24)^ Furthermore, up to 40% of family members and caregivers of critically ill patients appear to experience depressive symptoms up to 1 year after ICU discharge.^(55)^ Interventions such as extended family visitation^(56)^ and improved communication^(57)^ during the ICU stay can help reduce patients’ emotional burden.

### Physical limitations

The patient’s ability to function independently is commonly affected following hospitalization for a critical illness.^(28)^ For example, patients who have been treated for sepsis typically experience the development of 1 to 2 new limitations in activities of daily living (ADLs), such as the inability to manage finances, bathe, or use the toilet independently, in the months following hospital discharge.^(10)^ Patients often experience physical weakness due to critical illness, which can be attributed to myopathy, neuropathy, cardiorespiratory impairments, cognitive impairment, or some degree of combination of these conditions. Swallowing difficulties are also common and may arise from muscular weakness or neurological damage,^(10,29)^increasing the likelihood of aspiration,^(58)^ a common cause of rehospitalization.

Physical function generally has the potential to improve after hospital discharge.^(59)^ The six-minute walk test (6MWT) is a predictor of long-term physical improvement among ICU survivors. Compared with the initial assessment at 3 months, a significant improvement in the 6MWD is reported at 12 months. Female sex, preexisting comorbidities, and ARDS (*versus* non-ARDS) were associated with lower 6MWT results.^(60)^ The causes of this decline in functional capacity are multifactorial.^(59)^ However, it is important to note that physical function often remains below the normal levels seen in the general population and frequently does not fully return to the levels observed prior to ICU admission.^(10,29)^Additionally, survivors with greater functional dependence have higher mortality rates in the first years after hospital discharge,^(61,62)^and functional decline also appears to be a risk factor for the development of psychiatric syndromes after ICU discharge.^(48)^

### Cognitive decline

Patients admitted to the hospital for acute critical illness may experience neurological damage due to diverse mechanisms, including cerebral ischemia, hypoxia, metabolic disturbances, and neuroinflammation.^(12)^ Cognitive impairment is found in a significant number of critical illness survivors, with prevalences ranging from 10 to 71% after one year of follow-up, depending on the studied population.^(12)^ Common domains of cognition affected in the context of critical illness, including processing speed, memory, executive function (i.e., the ability to plan, focus attention, remember instructions, and deal with multiple tasks), and attention^(65,66)^variables, have been investigated as potential risk factors for long-term cognitive impairment following acute illness. The duration of delirium has been identified as a potential risk factor, although it remains unclear whether delirium is merely associated with cognitive impairment or is part of the causal pathway leading to persistent cognitive dysfunction. In addition to cognitive deterioration, delirium is also linked to a decline in the ability to perform instrumental ADLs, which encompass tasks such as medication management, arranging transportation, handling finances, and shopping for essential household items. Potential risk factors for delirium among critical care patients include sepsis, advanced age, and profound sedation.^(10,12)^Another potential risk factor for enduring cognitive impairment is a diminished cognitive reserve (CR). CR is a concept tied to the brain’s adaptability and is influenced by factors such as one’s lifestyle, education, and intellectual capacity. Cognitive reserve acts as a protective shield against the potential onset of dementia, predicts improved cognitive functioning in individuals with psychiatric disorders, and mitigates the risk of cognitive impairment after being discharged from an ICU.^(69,70)^

### Exacerbation and development of chronic medical conditions

Critical illness survivors frequently experience readmissions due to potentially treatable conditions in the outpatient setting.^(71)^ The most common reasons for readmission include the exacerbation of heart failure, acute worsening of chronic kidney disease, exacerbation of chronic obstructive pulmonary disease, and reinfections. It is plausible that patients with sepsis may have impaired balance due to organic dysfunctions (e.g., a decline in renal or respiratory function) or disruption of homeostatic mechanisms (e.g., blood pressure instability or fluid imbalance) induced by critical illness, which increases their susceptibility to the worsening of these chronic processes. Sepsis survivors appear to have a greater incidence of cardiovascular events and acute kidney injury.^(10,29)^The excess risk of late cardiovascular events (myocardial infarction, stroke, sudden cardiac death, or ventricular arrhythmias) may persist for at least 5 years following hospital discharge.^(72)^

### Reinfections

The rehospitalization of septic patients is mainly due to the recurrence of sepsis as well as pneumonia and urinary tract, skin or soft tissue infections.^(25,73,74)^The increased risk of infection in these patients occurs due to postsepsis syndrome, which encompasses various clinical manifestations, including metabolic changes, such as reduced total body protein, increased fluid retention, delayed return to normal hydration, and elevated total energy expenditure.^(75)^ Therefore, the short- and long-term pathophysiology of sepsis presents a multifaceted challenge, and the resolution of immune system changes postsepsis is intricate and often protracted, with many patients continuing to experience inflammatory changes, immune suppression, or both after sepsis.^(10)^

### Poor quality of life

Survivors of an acute critical care illness express a perception of experiencing a low QoL compared to the general population and encounter difficulties in returning to activities they engaged in before their ICU admission.^(76)^ Additionally, at 6 months posthospital discharge, approximately one-third of patients are unable to regain independent living.^(10,29)^

Preadmission comorbidities are associated with a significant reduction in QoL following critical illness.^(76)^ In septic patients, the severity of the infectious episode also acts as a marker for worsened QoL and mortality.^(77)^ Nevertheless, despite facing greater physical disability and an elevated incidence of pain, most patients are satisfied with their QoL and express a willingness to return to the ICU, if necessary, although many frequently recall unpleasant memories of their stay.^(14,78)^

### Inability to return to work and impairment of social relationships

Critical illness survivors grapple with long-term physical, psychological, and cognitive aftereffects, which impede their ability to reenter the workforce. Reentering the workforce is frequently regarded as the epitome of socioeconomic reintegration. Regardless of employment status before the critical illness or returning to work afterward, there are a range of social and financial challenges after hospital discharge, including financial strain resulting from existing debt; financial losses incurred during hospital admission; ongoing financial shortfalls; strain on relationships; caregiver burden, including social and financial strain for caregivers; loss of hobbies or interests; and social isolation. Ultimately, the possibility of significant changes in both social and financial circumstances can lead to a loss of identity, decreased self-esteem, and a reduced quality of life. The rates of returning to work varied, with percentages ranging from 21% to 49% at 3 months, 45% to 75% at 6 months, and 45% to 69% at 12 months.^(79)^ Psychosocial well-being is positively linked to the return-to-work process, including improved QoL and fewer depressive symptoms.^(80,81)^Conversely, those who do not return to work experience worsened cognitive function, psychological disorders, and more frequent hospitalizations.^(80)^ Among employed survivors, reduced working hours and job changes are common, and both employed and unemployed individuals face decreased income.^(79)^In addition, for cognitive impairment, it was determined that few patients had resumed driving within 1 month of discharge.^(82)^

Reconnecting with previous social circles can also prove to be quite challenging for ICU survivors. Larger groups of friends might not fully grasp the severity of the illness or the necessary recovery time, leading survivors to feel isolated and pressured to resume their previous activities. Certain hobbies or activities could be detrimental to their recovery; for instance, individuals with addictions may need to steer clear of situations that could trigger a relapse. Additionally, new physical limitations may prevent participation in certain sports or fitness activities, further restricting social interactions.^(31,83)^

## PREVENTION OF DISABILITIES DURING CRITICAL ILLNESS MANAGEMENT

After recovering from critical illness, many ICU survivors will experience one or more impairments;^(10,14)^however, scientific evidence for its prevention, although growing, remains limited. The prevention of PICS is now recognized as starting from the onset of critical illness,^(84)^and the most well-known modifiable factors associated with PICS are sedation, delirium, agitation, mechanical ventilation and length of stay.^(54)^Evidence-based interventions, such as minimizing iatrogenic harm, preventing, and managing delirium, early mobilization to prevent muscle wasting, and involving families, have demonstrated their effectiveness in reducing the numerous complications associated with critical illness. These interventions have been integrated into a comprehensive bundle of care called the ABCDEF Bundle, which represents significant advancements in preventing PICS and its consequences over the past decades. To address the growing number of impairments observed in critical illness survivors, the American College of Critical Care Medicine initially developed and updated the Prevention and Management of Pain, Agitation/Sedation, Delirium, Immobility, and Sleep Disruption in Adult Patients in the ICU (PADIS) Guidelines.^(85)^ Subsequently, the Society of Critical Care Medicine (SCCM) developed a large-scale quality improvement program utilizing these guidelines to create the ABCDEF Bundle, also known as the ICU Liberation Bundle, which focuses on addressing pain, agitation, and delirium in the ICU.^(86)^ The ABCDEF Bundle consists of evidence-based interventions that have been validated in multiple clinical trials. These components include (A) assessing, preventing, and managing pain; (B) implementing spontaneous awakening trials (SAT) and spontaneous breathing trials (SBT); (C) selecting appropriate analgesia and sedation options; (D) assessing, preventing, and managing delirium; (E) promoting early mobility and exercise; and (F) engaging and empowering family members (Table 2).^(16)^ Each component has a potential role in improving ICU outcomes and reducing the burden of PICS in survivors. By incorporating these interventions as part of a comprehensive care approach, the ABCDEF Bundle aims to optimize patient care and enhance long-term recovery. The combined impact of these interventions works synergistically to promote better outcomes and mitigate the adverse effects of critical illness in ICU survivors.^(16,87)^

**Table 2:** ABCDEF bundle elements and rationale

Bundle element	Rationale
A. Assess, prevent, and manage pain	With adequate pain control, many patients can tolerate mechanical ventilation without sedative infusions
B. Both SATs and SBTs	SATs and SBTs are associated with a faster liberation from mechanical ventilation and reduced mortality
C. Choice of sedation	Nonbenzodiazepine sedation is associated with lower rates of delirium compared to benzodiazepines
D. *Delirium*	*Delirium* is common and associated with increased mortality. Regular screening improves recognition
E. Early mobility and exercise	Early mobility is linked to a shorter duration of *delirium*
F. Family engagement and empowerment	Family involvement improves satisfaction and communication and reduces *delirium* rates

Source: Marra A, Ely EW, Pandharipande PP, Patel MB. The ABCDEF bundle in critical care. Crit Care Clin. 2017;33(2):225-43.^(16)^

SATs - spontaneous awakening trials; SBTs - spontaneous breathing trials.

It is common for patients with sepsis to experience new morbidities, such as weakness and cognitive impairment, as well as further health deterioration after hospitalization.^(10)^ However, currently, there is limited evidence available to guide the prevention of postsepsis morbidity or the management of patients who survive hospitalization for sepsis (Table 3).^(88-90)^

**Table 3:** In-hospital strategies to prevent postsepsis morbidity

Early critical ill care, mainly in sepsis	Early identification, broad-spectrum antibiotics, initial fluid bolus for patients with low systolic blood pressure or elevated lactate, and source control
Avoiding iatrogenic complications	The ABCDEF bundle
Deresuscitation and volume management	Positive fluid balance is associated with inability to ambulate, higher risk of development of multiple organ dysfunction, and discharge to a postacute care facility
Reconciling and titrating chronic medications	Medication lists are often adjusted at ICU discharge, and errors are common. Beyond reconciling a patient’s list of medications, clinicians must also consider the need for dosing adjustments
Avoiding aspiration	Aspiration following critical illness because of muscle weakness, cognitive impairment, and ongoing delirium. Bedside swallow evaluations may be helpful to assess swallow function. If the meal tray arrives hours later when patients are sleepy or confused, they may still be at high risk for aspiration
Avoiding reinfection	Strategies to reduce risk for secondary, hospital-acquired infection and infection-related readmission include stopping or narrowing antibiotics as soon as possible during the initial hospitalization, ensuring patients are up to date on recommended vaccines, counseling patients about their high risk for recurrent sepsis and the importance of seeking medical attention for signs or symptoms of infection, and having a lower threshold to evaluate and treat infectious signs and symptoms in recently septic patients
Tailoring nutrition	Critically ill patients often experience decreased appetite, an increased risk of aspiration, and delirium, which hinder adequate oral energy intake. Maintaining a hypercaloric and hyperprotein enteral nutrition supply, particularly after the first week of acute critical illness, can bring benefits in accelerating muscle recovery
Optimizing sleep	Sleep disruption is ubiquitous in patients admitted to the ICU. The implementation of chronobiological strategies (rhythmically occurring cues, such as reducing light, noise, feeding, temperature, activity, medical and nursing interventions, and sedatives), as well optimizing mechanical ventilation strategies, may facilitate sleep and recovery of postcritical illness

Source: Prescott HC. Preventing chronic critical illness and rehospitalization: a focus on sepsis. Crit Care Clin. 2018;34(4):501-13^(88)^; Ridley EJ, Lambell K. Nutrition before, during and after critical illness. Curr Opin Crit Care. 2022;28(4):395-400 ^(89)^; Showler L, Ali Abdelhamid Y, Goldin J, Deane AM. Sleep during and following critical illness: a narrative review. World J Crit Care Med. 2023;12(3):92-115.^(90)^

ICU - intensive care unit.

Intensive care unit diaries were provided by intensive care nurses with the aim of addressing patients’ psychological symptoms following critical illness and aiding in their recovery process.^(83)^ These diaries have been recognized as therapeutic tools, expressions of empathy and care, means of communication and orientation, supplements to follow-up visits, and humanizing elements in the ICU’s technical environment of the ICU.^(13,91,92)^The potential benefits of ICU diaries can be observed for patients, including improved well-being, enhanced QoL, better coping mechanisms, improved understanding of their illness, and reduced levels of anxiety and depression.^(13,83,91,93)^ Additionally, family members can experience improved well-being, better coping strategies, enhanced communication, reduced risk of PTSD, and decreased levels of anxiety and depression.^(83,91)^ Furthermore, ICU staff members may benefit from the use of ICU diaries through improved humanization of care, enhanced quality of care delivery, increased work satisfaction, and greater opportunities for reflection on critical care practices.^(83,91)^

## IDENTIFICATION OF PATIENTS AT AN INCREASED RISK FOR LONG-TERM DISABILITIES

After critical illness, it is common for patients who have survived to experience new or worsening impairments in physical, cognitive, and/or mental health function.^(10,13,14)^ However, it is essential to consider that not all individuals admitted to the ICU fall under the category of a high burden of impairments. Patients coming from the surgical theater, either due to major elective surgeries (e.g., cardiac surgeries, neurosurgeries, major lung resections, or complex abdominal surgeries) or minor surgical procedures performed on very frail or highly comorbid patients, as well as those admitted for acute coronary syndromes or requiring an ICU stay for a short duration (< 3 days), usually have a low risk of long-term impairment from critical illness and should not be characterized as individuals at high risk for long-term disabilities.^(22)^

**Figure 2:**
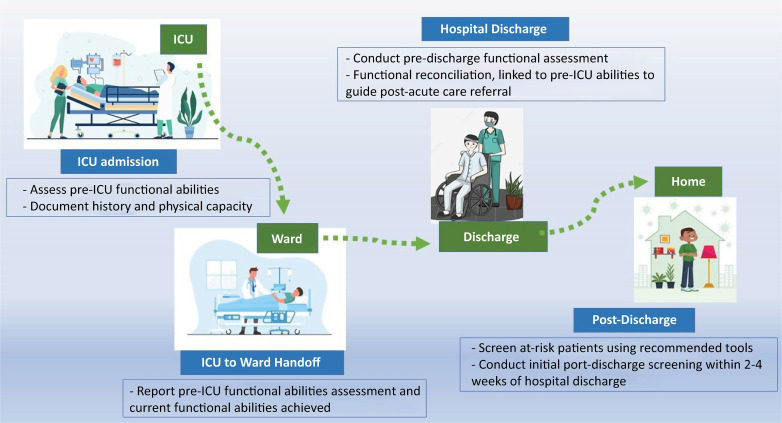
Recommended approach to functional assessments across the continuum of critical illness and recovery. ICU - intensive care unit.

Source: Mikkelsen ME, Still M, Anderson BJ, Bienvenu OJ, Brodsky MB, Brummel N, et al. Society of Critical Care Medicine’s International Consensus Conference on Prediction and Identification of Long-Term Impairments After Critical Illness. Crit Care Med. 2020;48(11):1670-9^(23)^.

A recent consensus conference by the SCCM on predicting and assessing PICS developed a document to identify high-risk patients (Table 4).^(23)^ The authors identified factors before (e.g., frailty, preexisting functional impairments), during (e.g., duration of delirium, sepsis, ARDS), and after (e.g., early symptoms of anxiety, depression, or posttraumatic stress disorder) critical illness that can be used to identify patients at high risk for cognitive, mental health, and physical impairments. They also emphasized the importance of pre-ICU functional assessments of patients because the patient’s preexisting functional status appears to have a greater impact on his/her ability to recover physically and cognitively than does the severity of acute critical illness (Figure 2).

**Table 4:** Patients at high risk for long-term cognitive, mental health, and physical impairments after critical illness (recommended screenings)

Functional domain	Before critical illness	During critical illness	After critical illness
Mental health	Preexisting mental health problems (anxiety, depression, or posttraumatic stress disorder)	Memories of frightening experiences in ICU	Early symptoms of anxiety, depression, or posttraumatic stress disorder
Physical	Preexisting functional disability Frailty Preexisting cognitive impairment		
Cognition	Preexisting cognitive dysfunction	Incidence and duration of delirium Sedation (benzodiazepines) Sepsis Shock Hypoxia Acute respiratory distress syndrome Life support (invasive mechanical ventilation)	

Source: Mikkelsen ME, Still M, Anderson BJ, Bienvenu OJ, Brodsky MB, Brummel N, et al. Society of Critical Care Medicine’s International Consensus Conference on Prediction and Identification of Long-Term Impairments After Critical Illness. Crit Care Med. 2020;48(11):1670-9.^(23)^

ICU - intensive care unit.

## PLANNING A SAFE DISCHARGE FROM THE INTENSIVE CARE UNIT AND FROM THE HOSPITAL

Safe discharge should not necessarily be interpreted as early discharge. Economic pressure on health care systems worldwide has led to a relentless pursuit of early discharge from ICUs and hospitals. However, current hospital admissions increasingly involve elderly individuals with comorbidities, fragility, and often a history of numerous prior hospitalizations and complex procedures (e.g., solid organ transplant, large tumor resection, chemotherapy for neoplasms) throughout their lives.^(94,95)^Despite the growing interest in early discharge hospital at-home services as a cost-effective alternative to inpatient care, current data reveal insufficient evidence supporting economic benefits, such as reduced hospital length of stay or improved health outcomes.^(96)^ Rushing can lead to imperfection. Hence, we are faced with the ongoing issue of hospital readmissions. Approximately 15 - 20% of hospitalized Medicare patients are readmitted to the hospital within 30 days after discharge,^(97,98)^and this occurs in more than 20% of septic patients.^(99)^In addition, premature discharge from the ICU and unplanned readmissions are associated with increased costs and prolonged hospital and ICU stays.^(25,100-102)^

However, the need for patient rehospitalization may solely reflect their greater clinical severity. Potential risk factors associated with an increased risk of acute care rehospitalization after discharge include advanced age, comorbidities, events that occurred during the initial hospitalization (such as the presence of delirium and duration of mechanical ventilation), and subsequent infections following hospital discharge.^(99,103)^ Currently, there are no adequate predictive models for rehospitalization.^(104)^ A recent meta-analysis^(101)^demonstrated that models relying solely on existing clinical risk or acuity scores, such as the Acute Physiology and Chronic Health Evaluation II (APACHE II), Sequential Organ Failure Assessment (SOFA), or Stability and Workload Index for Transfer (SWIT) score, performed poorly as predictors of rehospitalization or ICU readmission.^(105,106)^ Rehospitalization typically occurs due to infection.^(25,73,74)^ It is estimated that approximately 40% of hospital readmissions in septic patients may be potentially preventable.^(71)^Therefore, preventing unplanned hospital readmissions is a target for quality improvement, as readmissions can indicate unresolved acute illness, ongoing chronic disease, the development of new clinical problems, or gaps in outpatient care. However, previous government strategies aimed at reducing hospital readmissions^(107)^ have led to disastrous outcomes (increased mortality) in patients with pneumonia and heart failure.^(108-110)^

The transfer of patients from an ICU to a general hospital ward is a high-risk event, as it involves transitioning some of the most critically ill patients to a different health care environment and provider. Failures in care transition can be attributed to various factors and circumstances, including medication prescription errors, inadequate communication between ICU staff and ward staff, and a lack of coordination with other health care services.^(19)^ As a result, discharge planning becomes crucial to ensure seamless continuity of care, mainly for patients at the highest risk of readmission. In this context, critical care transition programs have shown promise in reducing the risk of ICU readmission among patients discharged from the ICU to a general hospital ward.^(21)^

During hospital discharge, clinicians should employ concise and standardized assessments, comparing the results with the patient’s pre-ICU functional abilities.^(23)^ This practice, known as “functional reconciliation,” is akin to the established concept of “medication reconciliation” and is recommended as a care coordination strategy to identify and address impairments throughout the continuum of care. The goal is to inform discharge decisions regarding the necessity of postacute care, such as long-term acute care facilities, skilled nursing facilities, inpatient rehabilitation, home health services, or outpatient rehabilitation. However, in this specific context, Shepperd et al.^(111)^ conducted a systematic review and reported that nursing discharge planning for older inpatients was associated with increased hospital length of stay. Nevertheless, their findings suggest that it does not effectively reduce the readmission rate or improve the QoL for older inpatients.

In the absence of validated prediction models, expert consensus has identified several potential risk factors that predict post-ICU impairments, including preexisting cognitive or physical impairment, mental health problems, delirium, sepsis, hypoxia, shock, benzodiazepine use, memories of frightening experiences in the ICU, and early symptoms of posttraumatic stress.^(23)^ The identification of these high-risk patients for hospitalization should guide care transition until better screening strategies are developed.

## FOLLOW-UP AFTER HOSPITAL DISCHARGE

To enhance long-term outcomes after critical illness, survivors advocate that health care providers adopt a broader disability framework when conducting post-ICU assessments. This approach should consider the individual’s prehospitalization health status, social determinants of health, and evolving goals.^(23)^ Survivors stress that for some, the path to recovery is ongoing, and learning to adapt to enduring impairments is a crucial part of rehabilitation. Instead of focusing solely on assessments at a single time point, a serial sustained assessment framework should prioritize repeated and dynamic evaluations aligned with significant patient-centered events, both expected and unexpected.

For patients at high risk, characterized as survivors with one or more potential risk factors according to table 3, the SCCM task force recommends serial assessments starting within 2 - 4 weeks of hospital discharge. These assessments can utilize screening tools such as the Montreal Cognitive Assessment (MoCA) test, Hospital Anxiety and Depression Scale (HADS), Impact of Event Scale-Revised (IES-6, for PTSD), 6-minute walk test, and/or the EuroQol-5D-5L, a measure of health-related quality of life and physical function (Table 5).^(23)^ However, the authors concluded that existing tools are insufficient to reliably predict PICS. Nonetheless, positive findings would prompt referrals for additional services and/or more comprehensive assessments upon transitioning out of the ICU (Figure 2).

**Table 5:** Recommended screening tools for detecting long-term cognition, mental health, and physical function

Domain	Screening test	Comments	Recommendation
Anxiety Depression	HADS HADS	A score of 8 or greater on the anxiety or depression subscale is used to identify symptoms of clinically significant anxiety or depression	Strong Strong
Posttraumatic stress disorder	IES-R or the abbreviated IES-6	The optimal screening threshold has been established as 1.6 (IES-R) or 1.75 (IES-6)	Weak
Physical function	6-min walk and/or EuroQol-5D-5L	Can be evaluated as a percent predicted against available normative data Includes assessments of mobility, self-care, and usual activities in addition to pain and anxiety/depression	Weak Weak
Cognition	Montreal Cognitive Assessment (MoCA)	Mild cognitive impairment defined as a score of 18–25, moderate as 10–17, and severe as less than 10	Strong

Source: Mikkelsen ME, Still M, Anderson BJ, Bienvenu OJ, Brodsky MB, Brummel N, et al. Society of Critical Care Medicine’s International Consensus Conference on Prediction and Identification of Long-Term Impairments After Critical Illness. Crit Care Med. 2020;48(11):1670-9.^(23)^

HADS - Hospital Anxiety and Depression Scale; IES-6 - Impact of Event Scale-6; IES-R - Impact of Events Scale-Revised.

Various strategies have been developed and implemented with varying levels of evidence to provide care for critically ill patients after hospital discharge (Table 6).^(112-120)^

**Table 6:** Strategies aimed at enhancing recovery in the postintensive care unit

Strategy	Potential favorable outcomes
ICU follow-up clinics	Reduction in early unplanned patient’s readmissions^(112)^ Reduction in morbidity and mortality among patients who received recommended postsepsis care^(113)^ Improvement in QoL of patients^(114)^ Improvement in patient satisfaction^(116)^ Reduction of ICU-staff burnout^(115)^
Peer support	Reduction of psychological morbidity^(117)^
Home-based care	Reduction in unplanned patient readmissions^(119)^
Improving transition to primary care	?^(118)^
Telehealth	Telerehabilitation interventions for stroke survivors have equal effects compared with face-to-face therapy^(120)^

benefit? Crit Care Explor. 2020;2(4):E0088^(116)^; Haines KJ, Beesley SJ, Hopkins RO, McPeake J, Quasim T, Ritchie K, et al. Peer support in critical care: a systematic review. Crit Care Med. 2018;46(9):1522-31^(117)^; Schmidt K, Worrack S, Von Korff M, Davydow D, Brunkhorst F, Ehlert U, et al. Effect of a primary care management intervention on mental health–related quality of life among survivors of sepsis: a randomized clinical trial. JAMA. 2016;315(24):2703-11^(118)^; Branowicki PM, Vessey JA, Graham DA, McCabe MA, Clapp AL, Blaine K, et al. Meta-analysis of clinical trials that evaluate the effectiveness of hospital-initiated postdischarge interventions on hospital readmission. J Healthc Qual. 2017;39(6):354-66^(119)^. Sarfo FS, Ulasavets U, Opare-Sem OK, Ovbiagele B. Tele-rehabilitation after stroke: an updated systematic review of the literature. J Stroke Cerebrovasc Dis. 2018;27(9):2306-18^(120)^.

### Post-intensive care unit follow-up clinics

One of the oldest and most widespread strategies for post-ICU care is the establishment of post-ICU follow-up clinics. Although no standardized model of post-ICU clinics has been rigorously evaluated or validated in trials, patients, families, and clinicians have identified several important elements of ICU follow-up care.^(22,83)^ These include addressing physical, cognitive, and emotional recovery through longitudinal assessments and goal setting by a multidisciplinary team, providing information on adapting to new impairments, offering peer support, implementing interventions tailored to caregivers, and providing guidance on welfare support and employment. Setting realistic expectations for recovery involves acknowledging uncertainty, providing a range of possible outcomes, and reassuring patients and families that the care team will continue to support them regardless of their outcome. However, which patients would benefit the most from an ICU follow-up clinic remains uncertain.^(22,78)^Patients who already receive comprehensive care from a multidisciplinary team, such as those undergoing cancer therapy or those who have undergone transplantation, may derive little additional benefit from a post-ICU clinic. Patients who are discharged to long-term care facilities or hospice are unlikely to attend outpatient services.

Given the prevalence of critical illness myopathy, cognitive dysfunction, and swallowing disorders in critically ill survivors, many post-ICU clinics incorporate physical therapists, occupational therapists, and speech therapists to evaluate patients or refer them for comprehensive evaluations by these specialists. The presence of a psychologist or psychiatrist is important for addressing preexisting psychiatric morbidity, considering that medications or therapies that previously stabilized such conditions may have been disrupted during the critical illness or subsequent transitions of care. In addition, given the high morbidity and mortality of the post-ICU population, the involvement of a palliative care specialist or the implementation of palliative care interventions and assessments is likely to have a positive impact.

### Peer support

Another strategy in the long-term care of critically ill patients is peer support. Peer support is the *“*process of providing empathy, offering advice, and sharing stories between ICU survivors. It is founded on the principles that both taking and giving support can be healing, if done with mutual respect*”*.^(121)^Peer support can take various forms, such as one-on-one peer-to-peer support for individuals with similar conditions working in partnership, support groups focused on behavioral changes and education, or the involvement of former patients in providing advice and support.^(83)^ When patients share their experiences and challenges with peers, it creates a safe environment where their journeys are normalized, and they no longer feel alone. This shared experience fosters a nonhierarchical reciprocal relationship, leading to successful peer support. Patients may be more receptive to accepting new behaviors and knowledge from peers who have “been there and done that” rather than professionals who may not share the same lived experience. Peer support also plays a vital role in providing hope for individuals affected by PICS. Through shared experiences, patients can witness how others further along in their recovery are coping and managing, despite starting from a similar baseline.

### Home-based care

Home-based care refers to the provision of health services directly to patients in their own homes, with support from trained health care professionals.^(83)^ The main objective is to offer guidance, assistance, and social support to individuals with significant health care needs, enabling them to maintain their independence as much as possible within their home environment. An important aim of home-based care interventions is to address the needs, values, and preferences of patients who are affected by multiple comorbidities, frailty, and disabilities. This care model is considered feasible as a health policy because it ensures cost-effectiveness while respecting the growing preference of many individuals to remain in their own homes rather than transitioning to residential care facilities.

### Telehealth

Telerehabilitation has become an integral component of health care delivery and is projected to continue expanding in the future, mainly after the COVID-19 pandemic. It has already demonstrated effectiveness in enhancing functionality and satisfaction for both patients and providers. Telerehabilitation is applicable across a broad spectrum of diagnostic areas within physical therapy treatment. It facilitates the evaluation and treatment of functional declines related to musculoskeletal, cardiovascular, pulmonary, neurological, and integumentary system disorders.^(122)^ Virtual physical therapy enables the utilization of diverse assessment and treatment approaches, delivering personalized remote care tailored to the individual’s unique requirements.

## CONCLUSION

In conclusion, the multifaceted challenges faced by survivors of critical care underscore the critical need for a comprehensive approach to their care. The extensive burden of physical, cognitive, and mental health impairments after intensive care unit discharge, along with increased vulnerability to adverse outcomes, including mortality and rehospitalization, cannot be overlooked. As discussed in this review, a proactive strategy involving early mobilization, delirium prevention, family presence and reduced sedation exposure during critical illness management holds promise for mitigating long-term disabilities. Equally important is the meticulous identification of patients at heightened risk for these disabilities, enabling tailored interventions. Furthermore, a well-structured plan for a safe intensive care unit and hospital discharge followed by robust posthospitalization follow-up focusing on rehabilitation and support, can contribute significantly to enhancing the quality of life for these survivors. In the era of modern critical care, prioritizing the anticipation of intensive care unit discharge and long-term follow-up for critical care patients is paramount, as it promises not only improved patient outcomes but also a more compassionate and holistic approach to recovery.
