# Supplementary material for: Unmasking the hidden aftermath: postintensive care unit sequelae, discharge preparedness, and long-term follow-up
Source: Crit Care Sci. 2024 May 27;36:e20240265en. doi: 10.62675/2965-2774.20240265-en (PMC11152445; doi:10.62675/2965-2774.20240265-en)
Supplement: Translation

# Translation: Desmascarando as consequências ocultas: sequelas pós-unidade de terapia intensiva, planejamento da alta e acompanhamento a longo prazo

**DOI:** 10.62675/2965-2774.20240265-pt

**Authors:** Cassiano Teixeira, Regis Goulart Rosa

**Affiliations:** 1 Brasil Departamento de Medicina Interna, Universidade Federal de Ciências da Saúde de Porto Alegre - Porto Alegre (RS), Brasil.; 2 Brasil Departamento de Medicina Interna, Hospital Moinhos de Vento - Porto Alegre (RS), Brasil.

**Keywords:** Estado terminal, Alta do paciente, Transição do hospital para o domicílio, Saúde mental, Cognição, Doenças cardiovasculares, Unidades de terapia intensiva

## Abstract

Parcela significativa de indivíduos que enfrentaram doença crítica sofre de síndrome pós-cuidados intensivos, caracterizada por comprometimento novo ou exacerbado da função física, cognitiva ou de saúde mental. Além disso, os sobreviventes geralmente apresentam maior risco de consequências adversas, como infecção, eventos cardiovasculares maiores, reinternação e taxas de mortalidade elevadas, durante os meses após a hospitalização. Esses achados reforçam a necessidade urgente de prevenção e manejo eficazes da deterioração da saúde a longo prazo no ambiente de cuidados intensivos. Embora haja poucas evidências conclusivas de ensaios clínicos randomizados bem desenhados, potenciais intervenções incluem estratégias como limitação da sedação, mobilização precoce, presença da família durante a internação na unidade de terapia intensiva, implementação de programas de transição multidisciplinares (da unidade de terapia intensiva para a enfermaria e do hospital para o domicílio) e acompanhamento especializado após a alta hospitalar. Esta revisão objetiva fornecer um resumo conciso da literatura médica recente sobre os desfechos a longo prazo após doenças críticas e destacar potenciais abordagens para prevenir e abordar a deterioração da saúde de sobreviventes de cuidados intensivos.

## INTRODUÇÃO

Nas últimas décadas, a organização sistemática das unidades de terapia intensiva (UTIs)^(1)^ desempenhou papel fundamental na obtenção de melhores resultados pelas equipes assistenciais.^(2)^ Com o uso racional de recursos^(3)^ e a protocolização da assistência,^(4,5)^ maior ênfase na humanização^(6,7)^ foi essencial para essa transformação, resultando em uma redução na morbimortalidade a curto prazo de pacientes críticos nos últimos anos.^(8,9)^ No entanto, essa redução na mortalidade trouxe à tona um novo desafio — a exacerbação de condições preexistentes ou o desenvolvimento de novas sequelas físicas,^(10,11)^ mentais^(12)^ e psicológicas^(13)^ entre os sobreviventes. Essas sequelas podem reduzir a qualidade de vida (QV), aumentar os gastos com saúde e aumentar o risco de reinternação e mortalidade a longo prazo.^(14)^

Recentemente, diretrizes de sociedades médicas abordaram a importância de melhorar os desfechos a longo prazo de sobreviventes de cuidados intensivos.^(15)^ Como resultado, esforços estão sendo feitos para implementar estratégias na UTI que previnam limitações a longo prazo;^(16)^ possibilitem o planejamento de alta segura para a enfermaria ou para o domicílio;^(17,18)^ fortaleçam a continuidade dos cuidados^(19)^ e reduzam a taxa de reinternações hospitalares e na UTI.^(20,21)^ Nesse contexto, as discussões avançaram de considerações teóricas para ações tangíveis no cuidado de pacientes críticos. Atualmente, a implementação de estratégias para o cuidado abrangente de pacientes críticos após a alta da UTI é uma prioridade,^(22)^ e essa mudança para uma abordagem holística pode contribuir para melhorar o bem-estar geral e os desfechos a longo prazo dos pacientes críticos e de seus familiares. Esta revisão refere-se exclusivamente a pacientes adultos; crianças não foram consideradas.

## DESFECHOS APÓS A ALTA DA UNIDADE DE TERAPIA INTENSIVA

Os pacientes que sobrevivem a uma doença crítica aguda apresentam nível significativo de morbidade^(23,24)^ e mortalidade^(25)^ nos meses seguintes à alta da UTI (Tabela 1). Esse risco é consideravelmente maior do que o observado em pacientes que não necessitam de cuidados intensivos^(26)^ e pode se manter elevado por vários anos após a alta hospitalar, especialmente em pacientes sépticos.^(27,28)^

**Tabela 1:** Sequelas da doença crítica

Condição	Consequências
Imunossupressão	As infecções são um dos principais motivos de reinternação de pacientes críticos, e uma parcela substancial poderia ser evitada. Além disso, a prevenção da reinternação parece reduzir a mortalidade dos pacientes a longo prazo
Fraqueza desenvolvida na UTI	Uso prolongado de ventilação mecânica, deambulação comprometida, prejuízo em AVDs, fraqueza muscular da faringe, dificuldades de deglutição, risco alto de aspiração, dificuldades profissionais e/ou redução da qualidade de vida relacionada à saúde por ≥ 5 anos
Comprometimento nutricional	Comprometimento da recuperação física e neurocognitiva
Neuropatia por aprisionamento	Pé ou punho caído, prejudicando a reabilitação e o funcionamento
Fragilidade	Incapacidade funcional, residência em casa de repouso, aumento da mortalidade pós-UTI
Disfunção cognitiva	Diminuição da atenção, concentração, velocidade de processamento, memória e/ou disfunção executiva por ≥ 5 anos; impacto profissional e no estado de saúde
Distúrbios psicológicos	Sintomas depressivos, ansiedade, TEPT, suicídio e/ou uso indevido de substâncias por ≥ 8 anos
Lesões por pressão	Presença prolongada pode impedir o retorno ao trabalho; estão associadas ao aumento da mortalidade pós-UTI
Risco de quedas	Quedas podem ser potencialmente devastadoras para pacientes debilitados, e sua prevenção (por exemplo, fortalecimento muscular e medidas educativas) pode reduzir significativamente a morbidade
Complicações orais	Gengivite, cárie, lesão ou perda dos dentes, exigindo acompanhamento odontológico a longo prazo
Endocrinopatias	Problemas de tireoide, função adrenal e do eixo hipotálamo-hipofisário, afetando a homeostase endócrina e a função sexual
Distúrbios musculoesqueléticos	Articulações “congeladas”, contraturas e ossificação heterotópica
Alterações na aparência	Alopecia, cicatrizes e desfiguração, afetando a reintegração social
Alterações no paladar	Dificuldades de alimentação e nutrição
Alterações auditivas ou visuais	Atraso na recuperação e no retorno ao trabalho e ao lar
Trauma relacionado ao procedimento	Lesão retal e uretral, disfunção das cordas vocais com fonação alterada, estenose de traqueia, dificultando as AVDs, reabilitação e retorno ao lar e ao trabalho
Disfunção renal	Comprometimento crônico da taxa de filtração glomerular, necessitando de terapia renal substitutiva, comprometendo a qualidade de vida relacionada à saúde e aumentando o uso de serviços de saúde
Doenças cardiovasculares	Sobreviventes de sepse e COVID-19 apresentam taxas mais altas de eventos cardiovasculares e tromboembólicos

Fonte: Herridge MS, Azoulay E. Outcomes after critical illness. N Engl J Med. 2023;388(10):913-24.^(24)^

Todas as condições coexistentes pós-UTI têm sido qualitativa ou quantitativamente associadas a prejuízos a longo prazo na qualidade de vida relacionada à saúde, à dificuldade de reintegração comunitária, ao retorno ao trabalho e ao aumento dos custos em saúde.

UTI - unidade de terapia intensiva; AVDs - atividades da vida diária; TEPT - transtorno de estresse pós-traumático.

### Maior mortalidade a longo prazo

Embora as taxas de mortalidade no ano seguinte à alta da UTI sejam geralmente altas, elas apresentam variação significativa de 7 a 59%.^(28-35)^ Pacientes que já foram internados em UTI apresentam taxas de mortalidade 7% mais altas após a alta hospitalar do que aqueles que não foram internados em UTI.^(26)^ Nos primeiros meses após a alta, metade das mortes desses pacientes é atribuída a complicações infecciosas, enquanto a outra metade está ligada a diversos fatores, como idade avançada, comorbidades preexistentes e mau estado funcional no momento da alta.^(10,25)^

Diversos fatores prévios à internação em UTI, como idade, comorbidades, sarcopenia e fragilidade,^(36-38)^ assim como a gravidade da doença aguda, medida pelo número e grau de disfunção orgânica^(28)^ e pelas complicações agudas na UTI (por exemplo: fraqueza muscular, dependência funcional e sintomas psicológicos),^(39)^ parecem contribuir de forma não linear para o aumento da mortalidade a longo prazo entre os sobreviventes. Em particular, disfunções neurológicas, como *delirium* e coma,^(40)^ e fraqueza muscular desenvolvida durante a permanência na UTI^(41)^ são as disfunções orgânicas agudas mais associadas à mortalidade a longo prazo.

### Comprometimento da saúde mental

A prevalência de diagnósticos psiquiátricos prévios é maior em pacientes críticos do que em coortes hospitalares e da população geral.^(13)^ Nos meses após a alta da UTI, aproximadamente 32 a 40% dos sobreviventes desenvolvem ansiedade;^(42)^ 29 a 34% desenvolvem depressão;^(43)^ e 16 a 23% desenvolvem transtorno de estresse pós-traumático (TEPT).^(44)^ Há aumento na ocorrência de novos diagnósticos psiquiátricos e no uso de medicamentos psicoativos nos meses seguintes à alta.^(45)^ No primeiro ano após a alta, quase 20% dos sobreviventes começam a usar novos medicamentos psicotrópicos (hipnóticos, antidepressivos, ansiolíticos ou antipsicóticos).^(46)^ Além disso, há aumento significativo no risco de autolesão e suicídio entre os sobreviventes de cuidados intensivos com transtornos psiquiátricos prévios.^(47)^ O sofrimento emocional e a morbidade psiquiátrica após doença crítica devem ser entendidos como um conjunto de síndromes que compartilham sintomas e potenciais fatores de risco, não como entidades completamente distintas.^(13)^ Os sintomas de TEPT incluem hiperatividade, lembranças intrusivas e comportamentos de evitação associados a um evento traumático. Os sintomas de depressão incluem humor deprimido, anedonia (incapacidade de sentir prazer) e sentimentos de culpa ou de inutilidade. Os sintomas de ansiedade incluem preocupação excessiva e sentimentos de medo ou ameaça percebida. Quando esses sintomas são significativos e impactam o funcionamento diário, pode-se considerar o diagnóstico de uma condição psiquiátrica.

A prevalência de condições psiquiátricas tende a aumentar ao longo do tempo após a alta da UTI,^(13)^ e muitos indivíduos apresentam uma combinação de síndromes psiquiátricas.^(13,48)^ Quanto mais síndromes psiquiátricas apresentadas por um sobrevivente, maior o impacto em sua QV^(48-52)^ e maior o risco de mortalidade após deixar a UTI.^(53)^ No entanto, identificar potenciais fatores de risco associados à doença mental entre sobreviventes é tarefa complexa e que envolve diversos fatores em diferentes domínios, como idade, condições psiquiátricas preexistentes, estresse emocional agudo e incapacidade física durante a internação na UTI^(13,48)^ (Figura 1). Os pacientes mais jovens estão mais propensos a desenvolver TEPT, e um histórico prévio de doença mental é um forte preditor de doença psiquiátrica relacionada à síndrome pós-cuidados intensivos (PICS - *post intensive care syndrome*) em pacientes e familiares.^(13,54)^

**Figura 1:** Os potenciais fatores de risco para morbidade psiquiátrica após alta da unidade de terapia intensiva incluem doença psiquiátrica preexistente e sintomas psiquiátricos durante a internação.(13,48) Os *emojis* representam a qualidade de vida dos pacientes de acordo com o número de morbidades psiquiátricas. UTI - unidade de terapia intensiva; TEPT - transtorno do estresse pós-traumático.

Fonte: Adapted from: Prince E, Gerstenblith TA, Davydow D, Bienvenu OJ. Psychiatric morbidity after critical illness. Crit Care Clin. 2018;34(4):599-608^(13)^; Teixeira C, Rosa RG, Sganzerla D, Sanchez EC, Robinson CC, Dietrich C, et al. The burden of mental illness among survivors of critical care—risk factors and impact on quality of life: a multicenter prospective cohort study. Chest. 2021;160(1):157-64^(48)^.

Familiares de pacientes na UTI também apresentam frequências elevadas de sintomas relacionados a ansiedade, depressão, TEPT e luto prolongado.^(24)^ Além disso, até 40% dos familiares e cuidadores de pacientes críticos podem apresentar sintomas depressivos até 1 ano após a alta da UTI.^(55)^ Intervenções como visitas familiares prolongadas^(56)^ e melhoria da comunicação^(57)^ durante a internação podem ajudar a reduzir a carga emocional dos pacientes.

### Limitações físicas

Após a internação por uma doença crítica, é comum que a capacidade dos paciente de funcionar de forma independente seja afetada.^(28)^ Por exemplo, pacientes tratados por sepse geralmente desenvolvem de uma a duas novas limitações em atividades da vida diária (AVDs), como incapacidade de gerenciar finanças, tomar banho ou usar o banheiro de forma independente, nos meses seguintes à alta hospitalar.^(10)^ Os pacientes frequentemente sentem fraqueza física devido à doença crítica, que pode ser atribuída a miopatia, neuropatia, comprometimentos cardiorrespiratórios, comprometimento cognitivo ou algum nível de combinação dessas condições. Dificuldade para engolir também é comum e pode ser causada por fraqueza muscular ou dano neurológico,^(10,29)^ aumentando a probabilidade de aspiração,^(58)^ uma causa comum de reinternação.

Geralmente, há potencial para melhorar a função física após a alta hospitalar.^(59)^ O teste de caminhada de 6 minutos (TC6M) é um preditor de melhoria física a longo prazo em sobreviventes da UTI. Em comparação à avaliação inicial aos 3 meses, uma melhoria significativa na distância percorrida no TC6M é relatada aos 12 meses. Sexo feminino, comorbidades preexistentes e síndrome do desconforto respiratório agudo (SDRA) (*versus* ausência de SDRA) foram associados a piores resultados no TC6M.^(60)^ As causas desse declínio da capacidade funcional são multifatoriais.^(59)^ No entanto, é importante ressaltar que a função física geralmente permanece abaixo dos níveis normais observados na população geral e, muitas vezes, não retorna completamente aos níveis observados antes da internação na UTI.^(10,29)^ Além disso, sobreviventes com maior dependência funcional têm taxas de mortalidade mais altas nos primeiros anos após a alta hospitalar,^(61,62)^ e o declínio funcional também parece ser um fator de risco para o desenvolvimento de síndromes psiquiátricas após a alta da UTI.^(48)^

### Declínio cognitivo

Pacientes internados por doença crítica aguda podem sofrer danos neurológicos devido a diversos mecanismos, como isquemia cerebral, hipóxia, distúrbios metabólicos e neuroinflamação.^(12)^ Observa-se comprometimento cognitivo em um número significativo de sobreviventes de doenças críticas, com prevalências que variam de 10 a 71% após o primeiro ano de acompanhamento, dependendo da população estudada.^(12)^ Domínios comuns da cognição afetados no contexto de doença crítica, como velocidade de processamento, memória, função executiva (ou seja, a capacidade de planejar, focar a atenção, lembrar instruções e lidar com várias tarefas) e variáveis de atenção,^(65,66)^ têm sido investigados como possíveis fatores de risco para comprometimento cognitivo a longo prazo após doença aguda. A duração do *delirium* foi identificada como potencial fator de risco, embora ainda não esteja claro se o *delirium* está apenas associado ao comprometimento cognitivo ou se faz parte do curso causal que resulta em disfunção cognitiva persistente. Além do declínio cognitivo, o *delirium* também está associado a um declínio na capacidade de realizar AVDs instrumentais, incluindo tarefas como administração de medicamentos, transporte, gerenciamento de finanças e compras de itens essenciais para a casa. Potenciais fatores de risco para *delirium* em pacientes críticos incluem sepse, idade avançada e sedação profunda.^(10,12)^ Outro possível fator de risco para comprometimento cognitivo persistente é a diminuição da reserva cognitiva (RC). A RC é um conceito ligado à adaptabilidade do cérebro, sendo influenciada por fatores como estilo de vida, educação e capacidade intelectual. Ela atua como um escudo contra o potencial início de demência, prediz uma melhoria no funcionamento cognitivo em indivíduos com transtornos psiquiátricos e reduz o risco de comprometimento cognitivo após a alta da UTI.^(69,70)^

### Desenvolvimento e exacerbação de doenças crônicas

Sobreviventes de doença crítica são frequentemente reinternados devido a condições potencialmente tratáveis no ambiente ambulatorial.^(71)^ Os motivos mais comuns de reinternação incluem exacerbação da insuficiência cardíaca, piora aguda da doença renal crônica, exacerbação da doença pulmonar obstrutiva crônica e reinfecções. É possível que pacientes sépticos apresentem desequilíbrios devido a disfunções orgânicas (por exemplo: declínio da função renal ou respiratória) ou disrupção dos mecanismos de homeostase (por exemplo: instabilidade da pressão arterial ou desequilíbrio de fluidos) pela doença crítica, o que aumenta a suscetibilidade dos pacientes à piora desses processos crônicos. Sobreviventes de sepse parecem ter maior incidência de eventos cardiovasculares e lesão renal aguda.^(10,29)^ O risco excessivo de eventos cardiovasculares tardios (infarto do miocárdio, acidente vascular cerebral, morte súbita cardíaca ou arritmias ventriculares) pode persistir por pelo menos 5 anos após a alta hospitalar.^(72)^

### Reinfecções

A reinternação de pacientes sépticos se deve principalmente à recorrência de sepse, bem como pneumonia e infecções do trato urinário, pele ou tecidos moles.^(25,73,74)^ O aumento do risco de infecção nesses pacientes ocorre devido à síndrome pós-sepse, que engloba diversas manifestações clínicas, incluindo alterações metabólicas, como redução da proteína total, aumento da retenção de líquidos, demora na recuperação da hidratação normal e aumento do gasto energético total.^(75)^ Portanto, a fisiopatologia a curto e longo prazo da sepse apresenta um desafio multifacetado. A resolução de alterações imunológicas pós-sepse é complexa e, muitas vezes, demorada; muitos pacientes continuam a apresentar alterações inflamatórias, imunossupressão ou ambas após a sepse.

### Baixa qualidade de vida

Sobreviventes de doenças críticas agudas relatam baixa QV em comparação à população geral e enfrentam dificuldades para retornar às atividades de que participavam antes da internação na UTI.^(76)^ Além disso, 6 meses após a alta hospitalar, aproximadamente um terço dos pacientes ainda não consegue se tornar independente.^(10,29)^

Comorbidades preexistentes estão associadas a uma redução significativa da QV após doença crítica.^(76)^ Em pacientes sépticos, a gravidade do episódio infeccioso também funciona como marcador de piora da QV e mortalidade.^(77)^ No entanto, apesar de enfrentar maior incapacidade física e incidência elevada de dor, a maioria dos pacientes está satisfeita com sua QV e se mostra disposta a retornar à UTI se necessário, embora muitos possuam memórias desagradáveis do período de internação.^(14,78)^

### Incapacidade de voltar ao trabalho e comprometimento das relações sociais

Os sobreviventes de doenças críticas lidam com repercussões físicas, psicológicas e cognitivas a longo prazo, o que impede sua reintegração ao mercado de trabalho. O retorno ao trabalho costuma ser considerado o ápice da reintegração socioeconômica. Independentemente da situação profissional antes da doença crítica ou se o paciente conseguiu retornar ao trabalho após a alta, há uma série de desafios sociais e financeiros a serem enfrentados, como dificuldades financeiras decorrentes de dívidas; perdas financeiras incorridas durante a internação hospitalar; défice financeiro persistente; tensão nos relacionamentos; ônus social e financeiro para os cuidadores; perda de *hobbies* ou interesses e isolamento social. No fim, a possibilidade de mudanças significativas nas circunstâncias sociais e financeiras pode ocasionar perda de identidade, diminuição da autoestima e redução da QV. As taxas de retorno ao trabalho variam de 21 a 49% aos 3 meses, 45 a 75% aos 6 meses e 45 a 69% aos 12 meses.^(79)^ O bem-estar psicossocial está positivamente ligado ao processo de retorno ao trabalho, incluindo melhoria da QV e diminuição dos sintomas depressivos.^(80,81)^ Por outro lado, indivíduos que não conseguem retornar ao trabalho sofrem piora da função cognitiva, transtornos mentais e hospitalizações mais frequentes.^(80)^ Entre os sobreviventes que se mantêm empregados, é comum haver redução da carga horária e mudanças de função, e tanto os empregados quanto os desempregados sofrem redução de renda.^(79)^ Além disso, em relação ao comprometimento cognitivo, observou-se que poucos pacientes voltaram a dirigir dentro de 1 mês após a alta.^(82)^

Reconectar-se com os círculos sociais também pode ser bastante desafiador para os sobreviventes da UTI. Grupos maiores de amigos podem não compreender por completo a gravidade da doença ou o tempo necessário para a recuperação, fazendo com que os sobreviventes se sintam isolados e pressionados a retomar suas atividades anteriores. Certos *hobbies* ou atividades podem ser prejudiciais à recuperação; por exemplo, dependentes químicos devem evitar situações que possam ocasionar uma recaída. Além disso, o desenvolvimento de novas limitações físicas pode impedir a participação em certos esportes ou atividades físicas, restringindo ainda mais as interações sociais.^(31,83)^

## PREVENÇÃO DE INCAPACIDADE DURANTE O MANEJO DE DOENÇAS CRÍTICAS

Após se recuperarem de uma doença crítica, muitos sobreviventes da UTI desenvolvem uma ou mais limitações;^(10,14)^ no entanto, embora crescentes, ainda há poucas evidências científicas que embasam estratégias de prevenção. Atualmente, reconhece-se que a prevenção de PICS deve ser iniciada já no começo da doença crítica,^(84)^ e os fatores modificáveis mais conhecidos associados à PICS são sedação, *delirium*, agitação, ventilação mecânica e tempo de internação.^(54)^ Intervenções baseadas em evidências, como minimização de danos iatrogênicos, prevenção e manejo de *delirium*, mobilização precoce para prevenir perda muscular e envolvimento familiar, demonstraram ser eficazes na redução das inúmeras complicações associadas à doença crítica. Essas intervenções foram integradas a um conjunto abrangente de cuidados chamado *ABCDEF Bundle*, que representa avanços significativos na prevenção de PICS e suas consequências nas últimas décadas. Para tratar do crescente número de limitações observadas em sobreviventes de doenças críticas, o *American College of Critical Care Medicine* desenvolveu inicialmente e atualizou as *Clinical Practice Guidelines for the Prevention and Management of Pain, Agitation/Sedation, Delirium, Immobility, and Sleep Disruption in Adult Patients in the ICU*.^(85)^ Subsequentemente, a *Society of Critical Care Medicine* (SCCM) desenvolveu um programa de melhoria de qualidade em grande escala utilizando essas diretrizes para criar o *ABCDEF Bundle*, também conhecido como *ICU Liberation Bundle*, com foco no manejo de dor, agitação e *delirium* na UTI.^(86)^

O *ABCDEF Bundle* consiste em intervenções baseadas em evidências que foram validadas em múltiplos ensaios clínicos. Seus componentes incluem (A) avaliação, prevenção e manejo da dor; (B) implementação de testes de despertar diário e respiração espontânea; (C) seleção adequada de analgesia e sedação; (D) avaliação, prevenção e manejo de *delirium*; (E) promoção de mobilização precoce e exercícios; e (F) envolvimento e capacitação dos familiares (Tabela 2).^(16)^ Cada componente tem um papel potencial na melhoria dos desfechos na UTI e na redução da carga de PICS em sobreviventes. Ao incorporar essas intervenções como parte de uma abordagem assistencial abrangente, o *ABCDEF Bundle* visa otimizar o cuidado dos pacientes e melhorar a recuperação a longo prazo. O efeito combinado dessas intervenções funciona de forma sinergética para promover melhores desfechos e mitigar os efeitos adversos da doença crítica em sobreviventes da UTI.^(16,87)^

**Tabela 2:** Elementos e justificativas do *ABCDEF Bundle*

Elemento	Justificativa
A. Avaliação, prevenção e manejo da dor	Com o controle adequado da dor, muitos pacientes conseguem tolerar ventilação mecânica sem infusões de sedativos
B. Despertar diário e TRE	O despertar diário e TRE estão associados a uma liberação mais rápida da ventilação mecânica e redução da mortalidade
C. Escolha de sedação	Sedação com não benzodiazepínicos está associada a taxas mais baixas de *delirium* em comparação à sedação com benzodiazepínicos
D. *Delirium*	O *delirium* é comum e está associado ao aumento da mortalidade. A triagem regular aumenta as chances de identificação
E. Mobilização precoce e exercício	A mobilização precoce está ligada a uma menor duração de *delirium*
F. Engajamento e empoderamento da família	O envolvimento familiar melhora a satisfação e a comunicação e reduz as taxas de *delirium*

Fonte: Marra A, Ely EW, Pandharipande PP, Patel MB. The ABCDEF bundle in critical care. Crit Care Clin. 2017;33(2):225-43.^(16)^

TRE - teste de respiração espontânea.

É comum que pacientes sépticos desenvolvam novas morbidades, como fraqueza e comprometimento cognitivo, bem como agravamento da saúde após a internação.^(10)^ No entanto, atualmente, há poucas evidências disponíveis para orientar a prevenção de morbidade pós-sepse ou o manejo de pacientes que sobreviveram à internação por sepse (Tabela 3).^(88-90)^

**Tabela 3:** Estratégias hospitalares para prevenir a morbidade pós-sepse

Cuidados intensivos precoces, principalmente em pacientes sépticos	Identificação precoce, antibióticos de amplo espectro, bólus inicial de líquidos para pacientes com pressão arterial sistólica baixa ou lactato elevado e controle do foco
Prevenção de complicações iatrogênicas	*ABCDEF Bundle*
“Derressuscitação” e manejo volêmico	O balanço hídrico positivo está associado à incapacidade para deambular, a maior risco de desenvolvimento de disfunção de múltiplos órgãos e à alta para um centro de cuidados pós-agudos
Reconciliação e titulação de medicamentos crônicos	As listas de medicamentos são frequentemente ajustadas na alta da UTI, e erros são comuns. Além da reconciliação medicamentosa, os médicos também devem considerar a necessidade de ajustes de dosagem
Prevenção de aspiração	Aspiração após doença crítica devido a fraqueza muscular, comprometimento cognitivo e *delirium* persistente. A avaliação funcional da deglutição à beira do leito pode ser útil. Se a comida chegar horas depois, quando os pacientes estiverem sonolentos ou confusos, eles ainda poderão correr alto risco de aspiração
Prevenção de reinfecção	As estratégias para reduzir o risco de infecção secundária adquirida no hospital e de reinternação relacionada à infecção incluem interromper ou reduzir o uso de antibióticos o mais rápido possível durante a hospitalização inicial, garantir que os pacientes estejam atualizados com as vacinas recomendadas, aconselhar os pacientes sobre seu alto risco de sepse recorrente e a importância de procurar atendimento em caso de sinais ou sintomas de infecção e adotar um limiar mais baixo para avaliar e tratar sinais e sintomas infecciosos em pacientes recentemente sépticos
Adaptação da nutrição	Pacientes críticos frequentemente apresentam diminuição do apetite, aumento do risco de aspiração e presença de *delirium*, o que dificulta a ingestão adequada de energia por via oral. Manter uma oferta de nutrição enteral hipercalórica e hiperproteica, principalmente após a primeira semana de doença crítica aguda, pode ajudar a acelerar a recuperação muscular
Otimização do sono	Os distúrbios do sono acometem todos os pacientes internados na UTI. A implementação de estratégias cronobiológicas (sinais que ocorrem ritmicamente, como redução de luz, ruído, alimentação, temperatura, atividade, intervenções médicas e de enfermagem e sedativos), bem como a otimização de estratégias de ventilação mecânica, podem facilitar o sono e a recuperação.

Fonte: Prescott HC. Preventing chronic critical illness and rehospitalization: a focus on sepsis. Crit Care Clin. 2018;34(4):501-13^(88)^; Ridley EJ, Lambell K. Nutrition before, during and after critical illness. Curr Opin Crit Care. 2022;28(4):395-400 ^(89)^; Showler L, Ali Abdelhamid Y, Goldin J, Deane AM. Sleep during and following critical illness: a narrative review. World J Crit Care Med. 2023;12(3):92-115.^(90)^

UTI - unidade de terapia intensiva.

Os diários de UTI foram elaborados por enfermeiros intensivistas com o objetivo de registrar os sintomas psicológicos dos pacientes após doenças críticas e auxiliar em seu processo de recuperação.^(83)^ Esses diários são reconhecidos como ferramentas terapêuticas, expressões de empatia e cuidado, meios de comunicação e orientação, complementos para consultas de acompanhamento e elementos humanizantes no ambiente técnico da UTI.^(13,91,92)^ Os potenciais benefícios dos diários de UTI para pacientes incluem melhora do bem-estar e da QV, desenvolvimento de melhores mecanismos de enfrentamento, compreensão aprofundada da doença e redução dos níveis de ansiedade e depressão.^(13,83,91,93)^ Além disso, os familiares também podem experienciar melhora do bem-estar e da comunicação; redução do risco de TEPT; redução dos níveis de ansiedade e depressão e desenvolvimento de melhores estratégias de enfrentamento.^(83,91)^ Já a equipe da UTI pode se beneficiar do uso desses diários por meio da melhoria da humanização do cuidado, do aprimoramento da qualidade da prestação de cuidados, do aumento da satisfação com o trabalho e de maiores oportunidades de reflexão sobre práticas de terapia intensiva.^(83,91)^

## IDENTIFICAÇÃO DE PACIENTES COM ALTO RISCO DE INCAPACIDADE A LONGO PRAZO

Após a doença crítica, é comum que a função física, cognitiva e/ou de saúde mental dos sobreviventes piore.^(10,13,14)^ No entanto, é preciso considerar que nem todos os indivíduos internados na UTI enfrentam o mesmo risco de desenvolver incapacidade. Pacientes provenientes do centro cirúrgico, seja devido a cirurgias eletivas de grande porte (cirurgias cardíacas, neurocirurgias, ressecções pulmonares ou cirurgias abdominais complexas) ou procedimentos cirúrgicos de pequeno porte realizados em pacientes muito frágeis ou altamente comórbidos, assim como aqueles internados por síndromes coronarianas agudas ou que necessitam de internação na UTI por um curto período (< 3 dias), geralmente têm baixo risco de desenvolver incapacidade a longo prazo devido à doença crítica e não devem ser caracterizados como indivíduos de alto risco para incapacidade a longo prazo.^(22)^

Recentemente, uma conferência de consenso científico da SCCM sobre predição e avaliação de PICS desenvolveu um documento para identificar pacientes de alto risco (Tabela 4).^(23)^ Os autores identificaram fatores antes (fragilidade, comprometimento funcional preexistente), durante (duração de *delirium*, sepse, SDRA) e após (sintomas iniciais de ansiedade, depressão ou TEPT) a doença crítica que podem ser utilizados para identificar pacientes com alto risco de comprometimento cognitivo, físico ou de saúde mental. Também enfatizaram a importância de realizar avaliações funcionais antes da internação na UTI, pois o estado funcional preexistente do paciente pode ter um impacto maior em sua capacidade de se recuperar fisicamente e cognitivamente do que a gravidade da doença crítica (Figura 2).

**Tabela 4:** Pacientes com alto risco de comprometimento cognitivo, físico e de saúde mental a longo prazo após doença crítica (avaliações recomendadas)

Domínio funcional	Antes da doença crítica	Durante a doença crítica	Após a doença crítica
Saúde mental	Problemas de saúde mental preexistentes (ansiedade, depressão ou transtorno de estresse pós-traumático)	Memórias de experiências assustadoras na UTI	Sintomas iniciais de ansiedade, depressão ou transtorno de estresse pós-traumático
Físico	Incapacidade funcional preexistente Fragilidade Comprometimento cognitivo preexistente		
Cognição	Disfunção cognitiva preexistente	Incidência e duração de *delirium* Sedação (benzodiazepínicos) Sepse Choque Hipóxia Síndrome respiratória aguda grave Suporte de vida (ventilação mecânica invasiva)	

Fonte:: Mikkelsen ME, Still M, Anderson BJ, Bienvenu OJ, Brodsky MB, Brummel N, et al. Society of Critical Care Medicine’s International Consensus Conference on Prediction and Identification of Long-Term Impairments After Critical Illness. Crit Care Med. 2020;48(11):1670-9.^(23)^

UTI - unidade de terapia intensiva.

**Figura 2:** Abordagem recomendada para avaliações funcionais ao longo da doença crítica e recuperação. UTI - unidade de terapia intensiva.

Fonte: Mikkelsen ME, Still M, Anderson BJ, Bienvenu OJ, Brodsky MB, Brummel N, et al. Society of Critical Care Medicine’s International Consensus Conference on Prediction and Identification of Long-Term Impairments After Critical Illness. Crit Care Med. 2020;48(11):1670-9^(23)^.

## PLANEJAMENTO DE ALTA SEGURA

Uma alta segura não deve ser necessariamente interpretada como precoce. A pressão econômica sobre os sistemas de saúde ao redor do mundo tem levado a uma busca incessante pela alta precoce das UTIs e dos hospitais. No entanto, atualmente, cada vez mais estão sendo internados idosos frágeis com comorbidades e, muitas vezes, histórico de diversas internações e procedimentos complexos (por exemplo, transplante de órgão sólido, ressecção de tumor grande, quimioterapia para neoplasias) ao longo de suas vidas.^(94,95)^ Apesar do crescente interesse em serviços de hospitalização domiciliar para pacientes com alta precoce como uma alternativa custo-efetiva à internação hospitalar, os dados atuais revelam falta de evidências que suportem benefícios econômicos, como redução do tempo de internação hospitalar ou melhora dos desfechos em saúde.^(96)^ A pressa pode levar à imperfeição. Portanto, estamos diante do problema contínuo das reinternações hospitalares. Aproximadamente 15 a 20% dos pacientes do Medicare são reinternados dentro de 30 dias após a alta,^(97,98)^ e isso ocorre em mais de 20% dos pacientes sépticos.^(99)^ Além disso, a alta precoce da UTI e as reinternações não planejadas estão associadas a um aumento nos custos e na permanência hospitalar ou na UTI.^(25,100-102)^

No entanto, a necessidade de reinternação pode apenas refletir uma maior gravidade clínica. Potenciais fatores de risco associados a um aumento do risco de reinternação em pacientes críticos após a alta incluem idade avançada, comorbidades, eventos ocorridos durante a hospitalização inicial (como *delirium* e duração da ventilação mecânica) e novas infecções.^(99,103)^ Atualmente, não há modelo preditivo adequado para reinternações.^(104)^ Metanálise recente^(101)^ demonstrou que modelos que utilizam apenas escores de risco clínico ou acuidade existentes, como o *Acute Physiology and Chronic Health Evaluation II* (APACHE II), *Sequential Organ Failure Assessment* (SOFA) e *Stability and Workload Index for Transfer* (SWIT), têm desempenho ruim na predição de reinternação hospitalar ou na UTI.^(105,106)^ As reinternações geralmente ocorrem devido a infecções.^(25,73,74)^ Estima-se que aproximadamente 40% das reinternações hospitalares de pacientes sépticos possam ser preveníveis.^(71)^ Desse modo, a prevenção de reinternações hospitalares não planejadas é alvo para a melhoria da qualidade, pois podem indicar doença aguda não resolvida, doença crônica em curso, desenvolvimento de novos problemas clínicos ou lacunas no atendimento ambulatorial. No entanto, estratégias governamentais anteriores para reduzir reinternações hospitalares^(107)^ tiveram resultados desastrosos (aumento da mortalidade) em pacientes com pneumonia e insuficiência cardíaca.^(108-110)^

A transferência de pacientes da UTI para uma enfermaria geral é um evento de alto risco, pois envolve movimentar alguns dos pacientes mais críticos para um ambiente hospitalar diferente com outros profissionais de saúde. Falhas na transição do cuidado podem ser atribuídas a vários fatores e circunstâncias, como erros na prescrição de medicamentos, comunicação inadequada entre a equipe da UTI e da enfermaria e falta de coordenação com outros serviços de saúde.^(19)^ Dessa forma, o planejamento da alta torna-se essencial para garantir a continuidade do cuidado de forma harmoniosa, principalmente para pacientes com maior risco de reinternação. Nesse contexto, os programas de transição de cuidados intensivos têm se mostrado promissores na redução do risco de reinternação na UTI entre os pacientes transferidos da UTI para uma enfermaria geral.^(21)^

Durante a alta hospitalar, os médicos devem realizar avaliações concisas e padronizadas, comparando os resultados com a capacidade funcional do paciente antes da internação.^(23)^ Essa prática, conhecida como “reconciliação funcional”, é semelhante ao conceito estabelecido de “reconciliação medicamentosa” e é recomendada como estratégia de coordenação de cuidados para identificar e abordar incapacidades ao longo do contínuo de cuidados. O objetivo é embasar, durante a alta, as decisões relativas à necessidade de cuidados pós-agudos, como Instituições de Longa Permanência, instalações de cuidados especializados, reabilitação hospitalar, atenção domiciliar ou reabilitação ambulatorial. No entanto, nesse contexto específico, Shepperd et al.^(111)^ conduziram uma revisão sistemática e relataram que o plano de alta para pacientes idosos internados estava associado a um aumento do tempo de permanência hospitalar. Todavia, os achados sugerem que isso não reduz de forma eficaz a taxa de reinternação nem melhora a QV dos pacientes idosos internados.

Na ausência de modelos preditivos validados, o consenso de especialistas identificou diversos potenciais fatores de risco que predizem incapacidade pós-UTI, como comprometimento cognitivo ou físico preexistente, problemas de saúde mental, *delirium*, sepse, hipóxia, choque, uso de benzodiazepínicos, memórias de experiências assustadoras na UTI e sintomas iniciais de TEPT.^(23)^ A identificação de pacientes com alto risco de hospitalização deve orientar a transição do cuidado até que estratégias de triagem melhores sejam desenvolvidas.

## ACOMPANHAMENTO APÓS A ALTA HOSPITALAR

Para melhorar os desfechos a longo prazo após uma doença crítica, os sobreviventes incentivam que os provedores de saúde considerem as avaliações pós-UTI dentro de uma estrutura de incapacidade mais ampla, que leve em conta o estado de saúde do paciente antes da internação, os determinantes sociais de saúde e os objetivos em evolução.^(23)^ Os sobreviventes destacam que, para alguns, o caminho para a recuperação é constante, e aprender a se adaptar a limitações permanentes é parte essencial da reabilitação. Em vez de priorizar avaliações realizadas em um único momento, uma estrutura de avaliação serial sustentada deve priorizar avaliações repetidas e dinâmicas alinhadas com eventos significativos centrados no paciente, tanto esperados quanto inesperados.

Para pacientes de alto risco, definidos como sobreviventes com um ou mais potenciais fatores de risco de acordo com a tabela 3, a força-tarefa da SCCM recomenda avaliações seriadas iniciando dentro de 2 a 4 semanas após a alta hospitalar. Essas avaliações podem utilizar ferramentas de triagem, como a *Montreal Cognitive Assessment* (MoCA), a *Hospital Anxiety and Depression Scale* (HADS), a *Impact of Event Scale-Revised* (IES-6, para TEPT), o TC6M e/ou *5-level EQ-5D version* (EQ-5D-5L), uma medida de QV relacionada à saúde e função física (Tabela 5).^(23)^ No entanto, os autores concluíram que as ferramentas existentes são insuficientes para predizer de forma confiável a ocorrência de PICS. Todavia, resultados positivos requerem que os pacientes sejam encaminhados para outros serviços e/ou avaliações mais abrangentes ao serem transferidos da UTI (Figura 2).

**Tabela 5:** Instrumentos de triagem recomendados para avaliar a cognição, saúde mental e função física a longo prazo

Domínio	Instrumento	Comentários	Recomendação
Ansiedade Depressão	HADS HADS	Uma pontuação de 8 ou mais na subescala de ansiedade ou depressão é usada para identificar sintomas de ansiedade ou depressão clinicamente significativa	Forte Forte
Transtorno de estresse pós-traumático	IES-R ou o IES-6 abreviado	O limiar ideal foi estabelecido como 1,6 para o IES-R e 1,75 para o IES-6	Fraca
Capacidade funcional	TC6M e/ou EQ-5D-5L	Pode ser avaliada como uma porcentagem prevista em relação aos dados normativos disponíveis Inclui avaliações de mobilidade, autocuidado e atividades habituais, além de dor e ansiedade/depressão	Fraca Fraca
Cognição	MoCA	Comprometimento cognitivo leve definido como pontuação de 18 a 25, moderado como 10 a 17 e grave como menos de 10	Forte

Fonte: Mikkelsen ME, Still M, Anderson BJ, Bienvenu OJ, Brodsky MB, Brummel N, et al. Society of Critical Care Medicine’s International Consensus Conference on Prediction and Identification of Long-Term Impairments After Critical Illness. Crit Care Med. 2020;48(11):1670-9.^(23)^

HADS – Escala de Ansiedade e Depressão Hospitalar; IES-R - *Impact of Events Scale-Revised*; IES-6 - *Impact of Event Scale-6*; TC6M – teste de caminhada de 6 minutos; EQ-5D-5L - *5-level EQ-5D version*; MoCA - *Montreal Cognitive Assessment.*

Diversas estratégias foram desenvolvidas e implementadas, com níveis diferentes de evidência, para a assistência de pacientes críticos após a alta hospitalar (Tabela 6).^(112-120)^

**Tabela 6:** Estratégias destinadas a melhorar a recuperação na unidade de terapia pós-intensiva

Estratégia	Potenciais desfechos favoráveis
Ambulatório pós-UTI	Redução da reinternação precoce não planejada^(112)^ Redução da morbidade e mortalidade de pacientes que receberam os cuidados pós-sepse recomendados^(113)^ Melhora da qualidade de vida dos pacientes^(114)^ Melhora da satisfação do paciente^(116)^ Redução de *burnout* entre a equipe da UTI^(115)^
Apoio de pares	Redução da morbidade psicológica^(117)^
Atenção domiciliar	Redução de reinternações não planejadas^(119)^
Melhorando a transição para a Atenção Primária	?^(118)^
Telemedicina	Intervenções de telerreabilitação para sobreviventes de AVC apresentam o mesmo efeito da terapia presencial^(120)^

Fonte: Bloom SL, Stollings JL, Kirkpatrick O, Wang L, Byrne DW, Sevin CM, et al. Randomized clinical trial of an ICU recovery pilot program for survivors of critical illness. Crit Care Med. 2019;47(10):1337-45^(112)^; Taylor SP, Chou SH, Sierra MF, Shuman TP, McWilliams AD, Taylor BT, et al. Association between adherence to recommended care and outcomes for adult survivors of sepsis. Ann Am Thorac Soc. 2020;17(1):89-97^(113)^; Rosa RG, Ferreira GE, Viola TW, Robinson CC, Kochhann R, Berto PP, et al. Effects of post-ICU follow-up on subject outcomes: a systematic review and meta-analysis. J Crit Care. 2019;52:115-25^(114)^; Haines KJ, Sevin CM, Hibbert E, Boehm LM, Aparanji K, Bakhru RN, et al. Key mechanisms by which post-ICU activities can improve in-ICU care: results of the international THRIVE collaboratives. Intensive Care Med. 2019;45(7):939-47^(115)^, McPeake J, Boehm LM, Hibbert E, Bakhru RN, Bastin AJ, Butcher BW, et al. Key components of ICU recovery programs: what did patients report provided UTI - unidade de terapia intensiva; AVC: acidente vascular cerebral.

### Ambulatório pós-unidade de terapia intensiva

Uma das estratégias mais antigas e disseminadas de acompanhamento pós-UTI são os ambulatórios pós-UTI. Embora nenhum modelo padronizado de ambulatório pós-UTI tenha sido rigorosamente avaliado ou validado em ensaios clínicos, pacientes, familiares e médicos identificaram diversos elementos importantes do acompanhamento pós-UTI.^(22,83)^ Entre esses elementos, estão a avaliação da recuperação emocional, cognitiva e física por meio de avaliações longitudinais e estabelecimento de metas por uma equipe multidisciplinar, e o fornecimento de informações sobre adaptação a novas limitações, apoio de pares, implementação de intervenções personalizadas para os cuidadores e orientação sobre apoio social e profissional. Estabelecer expectativas realistas para a recuperação envolve reconhecer as incertezas, informar a gama de possíveis desfechos e tranquilizar os pacientes e familiares de que a equipe continuará a apoiá-los, independentemente do desfecho. No entanto, ainda não se sabe quais pacientes se beneficiaram mais de um ambulatório pós-UTI.^(22,78)^ Pacientes que já recebem cuidados abrangentes de uma equipe multidisciplinar, como aqueles recebendo tratamento contra o câncer ou transplantados, podem obter poucos benefícios adicionais de um ambulatório pós-UTI. É improvável que pacientes transferidos para Instituições de Longa Permanência ou centros de cuidados paliativos utilizem serviços ambulatoriais.

Dada a prevalência de miopatia, comprometimento cognitivo e distúrbios de deglutição em sobreviventes de doença crítica, diversos ambulatórios pós-UTI contam com fisioterapeutas, terapeutas ocupacionais e fonoaudiólogos para avaliar o paciente ou referi-lo para avaliações mais abrangentes por esses especialistas. A presença de um psicólogo ou psiquiatra é importante para tratar a morbidade psiquiátrica preexistente, visto que os medicamentos ou tratamentos usados para estabilizar tais condições podem ter sido interrompidos durante a doença crítica ou a transferência do cuidado. Além disso, considerando a alta morbidade e a mortalidade entre a população pós-UTI, é provável que o envolvimento de um especialista em cuidados paliativos ou a implementação de intervenções e avaliações de cuidados paliativos tenham impacto positivo.

### Apoio de pares

Outra estratégia utilizada na assistência a longo prazo de pacientes críticos é o apoio de pares. O apoio de pares é o “processo de oferecer empatia, fornecer conselhos e compartilhar histórias entre sobreviventes da UTI. É fundamentado no princípio de que tanto receber quanto oferecer apoio pode ser curativo, se feito com respeito mútuo”.^(121)^ O apoio entre pares pode ocorrer de diversas formas: individual, em que duas pessoas com condições semelhantes trabalham em parceria; em grupos de apoio, com foco em mudanças comportamentais e educação; ou com o envolvimento de antigos pacientes que possam fornecer conselhos e apoio.^(83)^ Quando os pacientes compartilham suas experiências e desafios com seus pares, cria-se um ambiente seguro no qual suas jornadas são normalizadas, e eles não se sentem mais sozinhos. Essa experiência compartilhada fomenta uma relação recíproca sem hierarquia, promovendo apoio entre pares bem-sucedido. Os pacientes podem ser mais receptivos à adoção de novos comportamentos e conhecimentos de pessoas que já passaram pela mesma situação do que de profissionais que podem não compartilhar de mesma experiência. O apoio entre pares também desempenha papel essencial em fornecer esperança para pacientes afetados pela PICS. Por meio de experiências compartilhadas, os pacientes podem observar como outros, num estado de recuperação mais avançado, estão manejando e lidando com suas rotinas, apesar de terem tido um início semelhante.

### Atenção domiciliar

A atenção domiciliar se refere à prestação de serviços de saúde diretamente aos pacientes em seus próprios domicílios, com o apoio de profissionais de saúde treinados.^(83)^ O principal objetivo é oferecer orientação, assistência e apoio social a indivíduos com necessidades significativas de saúde, permitindo-lhes manter sua independência o máximo possível dentro de seu ambiente doméstico. Um objetivo importante da atenção domiciliar é atender às necessidades, aos valores e às preferências de pacientes afetados por múltiplas comorbidades, fragilidade e incapacidades. Esse modelo assistencial é considerado viável como política de saúde porque é custo-efetivo, ao mesmo tempo em que respeita a preferência de muitos pacientes de permanecer em suas próprias residências em vez de casas de repouso.

### Telemedicina

A telerreabilitação tornou-se componente essencial da prestação de serviços de saúde, e espera-se que continue se expandindo no futuro, principalmente após a pandemia da doença pelo coronavírus 2019 (COVID-19). Já demonstrou eficácia em aprimorar a funcionalidade e a satisfação tanto de pacientes quanto de profissionais de saúde. A telerreabilitação pode ser aplicada a uma ampla gama de áreas diagnósticas no contexto da fisioterapia. Facilita a avaliação e o tratamento de declínios funcionais relacionados a distúrbios do sistema musculoesquelético, cardiovascular, pulmonar, neurológico e tegumentar.^(122)^ A fisioterapia virtual permite a utilização de diversas abordagens de avaliação e tratamento, fornecendo cuidados remotos personalizados adaptados às necessidades únicas do indivíduo.

## CONCLUSÃO

Os desafios multifacetados enfrentados pelos sobreviventes de cuidados intensivos destacam a necessidade urgente de uma abordagem abrangente para seu cuidado. O ônus extensivo da incapacidade física, cognitiva e de saúde mental após a alta da unidade de terapia intensiva, com uma maior vulnerabilidade a desfechos adversos, incluindo mortalidade e reinternação, não pode ser negligenciado. Como discutido nesta revisão, uma estratégia proativa envolvendo mobilização precoce, prevenção de *delirium*, envolvimento familiar e redução da exposição à sedação durante o manejo da doença crítica pode mitigar a incapacidade a longo prazo. Igualmente importante é a identificação meticulosa de pacientes com alto risco para esses problemas, possibilitando intervenções personalizadas. Além disso, um plano bem-estruturado de alta segura da unidade de terapia intensiva e do hospital, seguido de um acompanhamento robusto com foco na reabilitação e no apoio, pode contribuir significativamente para a melhoria da qualidade de vida desses sobreviventes. Na era dos cuidados intensivos modernos, priorizar a antecipação da alta da unidade de terapia intensiva e o acompanhamento a longo prazo de pacientes críticos é primordial, visto que pode não apenas melhorar os desfechos do paciente, mas também permitir uma abordagem mais compassiva e holística para sua recuperação.
